# AI-Assisted Ultra-High-Sensitivity/Resolution Active-Coupled CSRR-Based Sensor with Embedded Selectivity

**DOI:** 10.3390/s23136236

**Published:** 2023-07-07

**Authors:** Mohammad Abdolrazzaghi, Nazli Kazemi, Vahid Nayyeri, Ferran Martin

**Affiliations:** 1 Electrical and Computer Engineering Department, University of Toronto, 10 King’s College Circle, Toronto, ON M5S3G4, Canada; mohammad.abdolrazzaghi@mail.utoronto.ca; 2 Electrical and Computer Engineering Department, University of Alberta, 116 St., Edmonton, AB T6G 2R3, Canada; nazli@ualberta.ca; 3 School of Advanced Technologies, Iran University of Science and Technology, Tehran 1684613114, Iran; nayyeri@iust.ac.ir; 4 Centro de Investigación en Metamateriales para la Innovación en Tecnologías Electrónica y de Comunicaciones (CIMITEC), Departament d’Enginyeria Electrònica, Universitat Autònoma de Barcelona, 08193 Bellaterra, Spain

**Keywords:** microwave sensor, coupled CSRR, active sensor, deep neural network, convolutional neural network, selectivity, material characterization, mixture sensing

## Abstract

This research explores the application of an artificial intelligence (AI)-assisted approach to enhance the selectivity of microwave sensors used for liquid mixture sensing. We utilized a planar microwave sensor comprising two coupled rectangular complementary split-ring resonators operating at 2.45 GHz to establish a highly sensitive capacitive region. The sensor’s quality factor was markedly improved from 70 to approximately 2700 through the incorporation of a regenerative amplifier to compensate for losses. A deep neural network (DNN) technique is employed to characterize mixtures of methanol, ethanol, and water, using the frequency, amplitude, and quality factor as inputs. However, the DNN approach is found to be effective solely for binary mixtures, with a maximum concentration error of 4.3%. To improve selectivity for ternary mixtures, we employed a more sophisticated machine learning algorithm, the convolutional neural network (CNN), using the entire transmission response as the 1-D input. This resulted in a significant improvement in selectivity, limiting the maximum percentage error to just 0.7% (≈6-fold accuracy enhancement).

## 1. Introduction

Microwave sensors, when juxtaposed with their optical and electrochemical equivalents, bring to the table a plethora of benefits. Notable amongst these are their compactness, ease of portability, the ability to perform label-free detection, CMOS/PCB compatibility [1,2], and paramountly, the feature of noncontact sensing [3,4,5,6,7,8,9]. These compact, cost-effective designs can deliver a high level of accuracy and sensitivity [3], making them highly attractive for a wide range of applications with various readout modes including single-/multi-frequency variation [10], amplitude/power variation [11], and phase variation [12,13,14] monitoring. Over the course of the last two decades, the use of microwave sensors has been witnessing a steady expansion in a diverse array of fields. These include but are not limited to biosensing [15,16,17,18,19,20], food quality monitoring [21], material characterization [22,23,24,25], environmental monitoring [26,27,28,29], as well as the monitoring of mechanical motion and strain [30]. The functioning of microwave-resonator-based sensors hinges on the environmental influences on the resonance profile of the sensor.

Planar sensors, in particular, due to their exposure of sensitive regions to the surrounding medium, are subject to changes in the ambient environment. Enhancing the sensitivity of resonator-based sensors makes them a fitting replacement for traditional, expensive equipment used for detecting minute variations. Some works in the literature for sensitivity enhancement include metamaterial SRR [31], branch line couplers [32], coupled-line couplers [33,34], and directional couplers [35]. In this work, conventional CSRRs are coupled together to exploit the sensitive region of the coupled CSRRs (CCSRR). The sensor sensitivity enhancement with respect to a single CSRR is investigated in this work. This improved sensitivity mainly comes from disturbing the coupling mechanism, while the propagation and interaction of the electromagnetic field with the surrounding medium are the underlying principles of microwave sensing.

While this characteristic is a major driver enabling noninvasive sensing, it also leads to a natural loss of power in free space [36,37,38,39]. The majority of these sensors, which operate passively without the utilization of electronics, are prone to typical dielectric and ohmic losses. This leads to a moderate quality factor, thereby limiting the performance of passive sensors due to low-quality factor resonances. To address this concern, a method has been proposed that involves augmenting the sensor with amplifying electronics to make up for the power loss via a recovery scheme, has been extensively researched as in [15,40]. In this approach, the passive core resonator is integrated into a regenerative amplifier or linked to a negative resistor. The negative resistor samples the sensor signal at resonance and returns it in an amplified form, with a modified phase that leads to constructive summation. The net effect of this process is the mitigation of the loss incurred from various sources, thereby enhancing the performance in sensitive applications. Through this active design, the quality factor can be amplified by orders of magnitude, enabling the achievement of ultra-high-resolution sensing. Particularly discussed in this article is the method to enable loss-compensation to coupled resonators, which allows the tuning of the high-resolution performance to a desired resonance.

The noncontact-sensing feature of microwave sensors allows the interrogation of materials in the vicinity of the sensor without direct contact [41]. This capability is advantageous as it facilitates the operation of the sensor in harsh environments [42,43,44,45] and inaccessible areas, thereby reducing maintenance costs and enhancing the lifespan of the sensor. However, this scheme presents challenges when the material under test (MUT) is situated among other materials or when an interfering medium or material is present within the sensor’s range, which can distort the measurement results. The principle of microwave sensing in resonator-based sensors hinges on the effective dielectric load on the resonator. It is evident that the combination of multiple liquids alters the dielectric properties, quantifiable using the Maxwell–Garnett equation [46,47], compared to a single-MUT. This article discusses one of the prevalent issues faced by microwave sensors—their sensitivity to the effective medium as opposed to a specific MUT—and proposes a machine-learning-based solution to this problem. In a complex matrix comprising two MUTs, the contribution of each material can be determined using a continuous regression model of the sensor response [48,49]. This regression can be computed readily when the background medium is known. However, when the target MUT is mixed with another unknown liquid, it becomes challenging to identify the concentration of the desired MUT. To address this, we propose a multi-feature sensor response, enhanced with a machine learning algorithm, to quantify the level of a chosen MUT in a mixture. These features could be frequency of resonance (F), resonance amplitude (A), and quality factor at resonance (Q). This resonance-based process results in a sensor that is not only sensitive but also selective to a single-MUT, a feature that is not inherently present in microwave sensors.

In the recent literature, machine learning has impacted the sensor data processing impressively towards performance enhancement with embedding features such as robustness [50], environmental noise cancellation [51], resolution enhancement in measured datapoints [52], and other applications in analyzing/optimizing sensor data [53,54,55]. To distinguish various concentrations of the desired MUT, we employed deep neural network (DNN) to map the sensor response features (FAQ) to the volume of each constituent liquid solvent. In this study, it is shown that for three common chemicals of water, ethanol, and methanol, DNN results in high accuracy for binary mixtures. Therefore, to expand the application of microwave sensors to three (and more) ingredients, a more sophisticated machine learning algorithm is used as a convolutional neural network (CNN) [56]. In this work it is shown that feeding the sensor with more datapoints taken from the sensor response using CNN enables this AI-assisted sensor to work with high accuracy. Even though a CNN typically requires 2D (image) [52] datapoints, data processing of images is resource intensive and time-consuming. It is shown that 1D sensor transmission response is enough to enable effective characterization of the MUT for any arbitrary binary/ternary mixture. This is the first contribution elaborating on the deep neural networks (DNN/CNN) being extensively utilized towards enabling the selectivity in microwave mixture sensing.

In this article, one of the main objectives is to develop and explain the main principle of coupling between resonators as given in Section 2. Then, the next main principle of operation of the proposed sensor is discussed as regenerative amplification in Section 3. These are the main motivations of this paper that make it unique due to simultaneous incorporation of both properties. Next, the sensor implementation based on these principles is manifested in Section 4 and Section 5. The sensor performance then is examined with respect to material sensing using a limited number of sensor data with a DNN as well as a large number of datapoints with CNN in Section 6.

## 2. Coupled CSRR Principle

**Figure 1:**
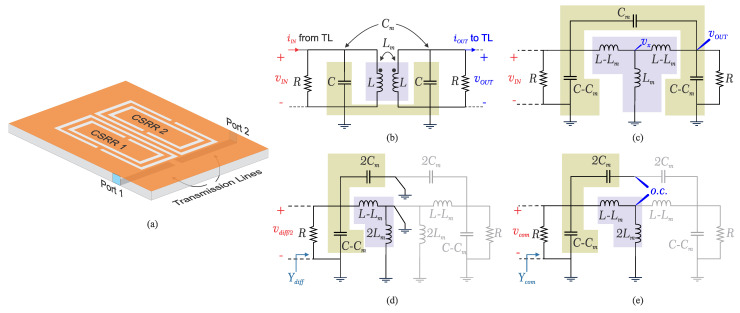
(**a**) Schematic of coupled CSRR. (**b**) Coupled resonator modeled by a parallel RLC model. (**c**) Simplified circuit considering the equivalent coupling capacitors/inductors. (**d**) Odd mode analysis. (**e**) Even mode analysis.

In the present study, we propose a coupled resonator structure designed for sensing applications, as depicted in Figure 1a. To elucidate the sensing functionality of this configuration, we begin by examining the fundamental characteristics of the structure and the manner in which their interactions contribute to sensing capabilities. In this context, two identical microwave complementary split-ring resonators are employed, which are configured in a coupled arrangement. A simplified circuit diagram of the proposed structure is illustrated in Figure 1b. Here, both resonators are depicted as parallel RLC components (representing resistance, inductance, and capacitance) [57,58], interconnected through a coupling capacitor ($C_{m}$) and a coupling inductor ($L_{m}$). This structure can be further simplified by infusing the coupling capacitors/inductors into the main structure as shown in Figure 1c. In order to find the resonance frequencies of the sensor, two approaches are presented with respect to nodal analysis based on Kirchhoff’s current law (KCL) as well as even/odd mode analysis as follows.

### 2.1. Nodal Analysis

The equivalent circuit model is analyzed to obtain the resonance frequencies of the coupled CSRR design. Applying KCL at three nodes of $v_{IN}=V_{1},\;v_{OUT}=V_{2},\;v_{x}=V_{3}$, as shown in Figure 1c, with respect to a current source *I* at the input results in:

(1) $$\begin{array}{cc} I & =V_{1}j\omega \left(C-C_{m}\right)+\frac{V_{1}-V3}{j\omega (L-L_{m})}+\left(V_{1}-V_{2}\right)jC_{m}\omega \end{array}$$

(2) $$\begin{array}{cc} 0 & =\frac{V_{3}-V_{1}}{j\omega (L-L_{m})}+\frac{V_{3}}{j\omega L_{m}}+\frac{V_{3}-V_{2}}{j\omega (L-L_{m})} \end{array}$$

(3) $$\begin{array}{cc} 0 & =\frac{V_{2}-V3}{j\omega (L-L_{m})}+j\omega \left(C-C_{m}\right)V_{2}+\left(V_{2}-V_{1}\right)j\omega C_{m} \end{array}$$

which can be summarized as follows:

(4) $$\underset{I}{\underset{︸}{\left[\begin{array}{c} I\\ 0\\ 0 \end{array}\right]}}=\underset{Y}{\underset{︸}{\left[\begin{array}{ccc} Y_{11} & Y_{12} & Y_{13}\\ Y_{21} & Y_{22} & Y_{23}\\ Y_{31} & Y_{32} & Y_{33} \end{array}\right]}}\underset{V}{\underset{︸}{\left[\begin{array}{c} V_{1}\\ V_{2}\\ V_{3} \end{array}\right]}}$$

Solving this system of linear equations results in the input admittance $Y_{11}$, from which the resonance frequencies of the coupled structure are extracted by applying $Im(Y_{11})=0$. This results in two distinct frequencies $\omega_{1}$ and $\omega_{2}$ as follows:

(5) $$\begin{array}{cc} \omega_{1} & =1/\sqrt{\left(L-L_{m}\right)\left(C+C_{m}\right)} \end{array}$$

(6) $$\begin{array}{cc} \omega_{2} & =1/\sqrt{\left(L+L_{m}\right)\left(C-C_{m}\right)} \end{array}$$

### 2.2. Even/Odd Mode Analysis

These quantities could also be extracted by inspecting the circuit with respect to odd and even modes, as shown in Figure 1d,e, respectively. It is helpful to consider the parasitic capacitance $C_{m}$ between the two resonators as a series combination of two capacitors sized as $2C_{m}$. Considering the odd mode, node $v_{x}$ becomes grounded, which using the first half of the circuit reveals the input admittance of

(7) $$Y_{diff}=\frac{1}{R}+j\omega \left(C-C_{m}\right)+j\omega \left(2C_{m}\right)+\frac{1}{j\omega (L-L_{m})}$$

which results in $Im(Y_{in-Odd})=0$ at

(8) $$\omega_{1}=1/\sqrt{\left(L-L_{m}\right)\left(C+C_{m}\right)}$$

The same analogy holds true with respect to even mode of analysis. Therefore, $v_{x}$ acts as an open circuit, which results in input admittance as follows:

(9) $$Y_{com}=\frac{1}{R}+j\omega \left(C-C_{m}\right)+\frac{1}{j\omega \left(L-L_{m}\right)+j\omega \left(2L_{m}\right)}$$

This leads to the second resonance frequency at $Im(Y_{in-Even})=0$ as follows:

(10) $$\omega_{2}=1/\sqrt{\left(L+L_{m}\right)\left(C-C_{m}\right)}$$

Consequently, the pair of identically coupled resonators can be conceptualized as a novel system consisting of two noncoupled resonators, each possessing distinct frequencies ($\omega_{1},\omega_{2}$). The modified resonances inherently encapsulate the coupling information, thus facilitating their integration into subsequent analyses. Furthermore, the entire design is connected to both the input and output transmission lines (TLs) via coupling capacitors that are not explicitly shown for brevity.

## 3. Regenerative Amplification

**Figure 2:**
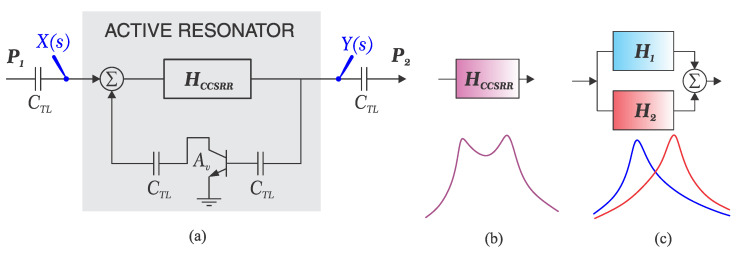
(**a**) Block diagram of regenerative amplifier for active resonator, (**b**) Equivalence of the coupled CSRR with a dual-band transmission profile, (**c**) Equivalence of the coupled CSRR with summation of two CSRR blocks.

In order to investigate the impact of the loss-compensating element such as amplifying transistors, it is helpful to reconsider the coupled structure in frequency domain with equivalent transfer function as shown in Figure 2a. The existing network incorporates a pair of discrete back-to-back resonators, configured in parallel since two resonance frequencies are distinct and the transmission response peaks at both frequencies $\omega_{1}$ and $\omega_{2}$. The corresponding transfer function representations for each resonator, as shown in Figure 2b,c, are delineated as follows:

(11) $$H_{1}\left(s\right)=\frac{K_{1}\frac{s\omega_{1}}{Q_{1}}}{s^{2}+\frac{s\omega_{1}}{Q1}+\omega_{1}^{2}},\;H_{2}\left(s\right)=\frac{K_{2}\frac{s\omega_{2}}{Q_{2}}}{s^{2}+\frac{s\omega_{2}}{Q1}+\omega_{2}^{2}}$$

In this context, $K_{1}$ and $K_{2}$ denote the maximum individual transmission amplitudes, while $\omega_{1}$ and $\omega_{2}$. represent the respective resonance frequencies. Additionally, $Q_{1}$ and $Q_{2}$ correspond to the loaded quality factors of each resonator. Upon activation of the amplifier integrated into the circuit, the predominant factors contributing to resonator loss, often termed as ohmic loss, are effectively attenuated. The amplifier, integrated into the feedback loop, introduces supplementary power into the system in a constructive manner, thereby augmenting the loaded quality factor. A summary of this system as shown in Figure 2a is provided as follows:

(12) $$\frac{Y(s)}{X(s)}=\frac{H_{1}+H_{2}}{1-A_{v}\left(H_{1}+H_{2}\right)}$$

Inspecting (12) at $\omega_{1}\pm \Delta \omega$, where $\Delta \omega \ll \omega_{1}$, the steep slope of the resonance skirt from $\omega_{2}$ oppresses its contribution close to $\omega_{1}$, which leads to the dominance of $H_{1}$, therefore $H_{1}+H_{2}\approx H_{1}$. As a result, total transfer function of the system close to $\omega_{1}$ is predominantly influenced by the amplifier $A_{v}$ in the feedback path as follows:

(13) $$\frac{Y(s)}{X(s)}\approx \frac{H_{1}}{1-A_{v}H_{1}}=\frac{\frac{K_{1}}{\left(1-A_{v}K_{1}\right)}\frac{\left(1-A_{v}K_{1}\right)}{Q_{1}}s\omega_{1}}{s^{2}+s\omega_{1}\frac{\left(1-A_{v}K_{1}\right)}{Q_{1}}+\omega_{1}^{2}}$$

It is evident that the original quality factor $Q_{1}$ as well as the amplitude of transmission $K_{1}$ are both enhanced by a factor of ${\left(1-A_{v}K_{1}\right)}^{-1}\gg 1$, assuming that $A_{v}<1/K_{1}$ to ensure the circuit stability. This represents how the sharpness and the maximum transmitted power are enhanced in a regenerative amplifier configuration, while the frequency of resonance remains intact. The second frequency band ($\omega_{2}$) also follows the same theory showing how the coupled resonators behave with respect to different features of their profile.

The amplification coefficient is governed by the active circuit, which is actuated utilizing the bias voltage. The operational principle of the regenerative amplifier is contingent upon two primary factors. Firstly, the system gain, denoted as $A_{v}$, must be sufficiently elevated to counterbalance the transmission loss incurred by the two series blocks, $H_{1}$ and $H_{2}$. Secondly, the signal phase necessitates an alteration of even multiples of 2π to ensure a constructive interaction when the existing signal in the loop converges with the input signal. Within the amplifier, the signal’s phase experiences a negative sign transformation. To accommodate the remaining phase modifications, supplementary TLs are employed, connecting the core passive resonator to the amplifier.

### The Frequency of Choice

**Figure 3:**
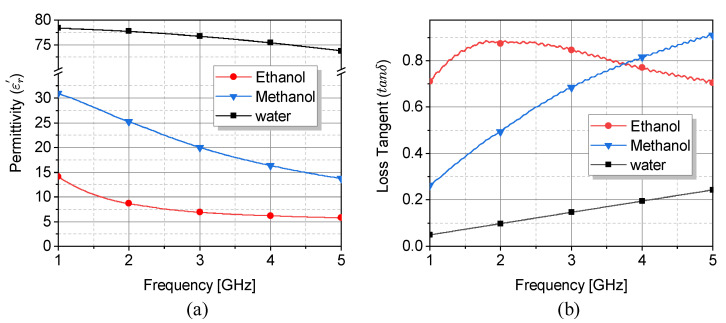
(**a**) The permittivity and (**b**) loss tangent of water, ethanol, and methanol using Debye function.

The operational frequency exerts distinct influences on the penetration depth into free space and the sensor’s sensitivity. Regarding sensitivity, the differences between MUTs at different frequencies are studied. Most materials of significant practical use exhibit linear, isotropic properties and possess negligible magnetic responses. In these instances, a scalar complex relative permittivity function is employed for effective modeling. When addressing the frequency dependence of electric permittivity, the widely-accepted single-pole Debye equation is often utilized as follows [15,51]:

(14) $$\varepsilon \left(\omega \right)=\omega_{\infty }+\frac{\varepsilon_{s}-\varepsilon_{\infty }}{1+j\omega \tau_{0}}$$

where the variables $\varepsilon_{s}$ and $\varepsilon_{\infty }$ represent the real components of the complex relative permittivity at static and infinite frequencies, respectively. Furthermore, $\tau_{0}$ denotes the relaxation time, which exhibits a direct relationship with the relaxation frequency. The relaxation frequency corresponds to the frequency at which both the imaginary component and the first derivative of the real component attain their maximal values. This model is accurate for the frequency span of interest, wherein only a single pole is present in the dispersive model of dielectric permittivity. Detailed observation of the dielectric properties of ethanol, methanol, and water is shown in Figure 3 with respect to the Debye parameters given in Table 1 [59], which demonstrates that the difference between the permittivity of water and ethanol/methanol increases at higher frequencies, leading to a high sensitivity. Therefore, a comparatively elevated frequency is chosen to enhance sensitivity [3,60]. Moreover, an inverse relationship exists between the resonator’s dimensions and the operational frequency mainly due to the penetration depth $\delta ={\left(\pi f\mu \sigma \right)}^{-1}$, where f,μ,σ are frequency, permeability and conductivity of MUT, respectively. As a result, the frequency of operation for this structure is chosen to be within 2–3 GHz.

## 4. Sensitivity and Resolution Enhancement

**Figure 4:**
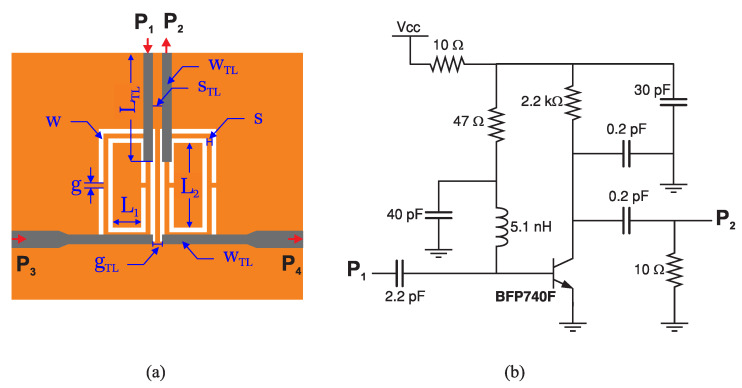
(**a**) Layout of the proposed CCSRR. (**b**) The proposed active circuit for loss compensation in a regenerative amplification format.

The proposed structure is designed in with microstrip technology using two identical CSRR, as shown in Figure 4. These CSRRs are fabricated by etching the solid ground, resulting in a defected ground. The slots are designed to be 0.5 mm thick, a dimension that can be accurately achieved using an in-house etching process with ammonium persulfate on a 0.8 mm thick RO4003 substrate with permittivity of 3.55 and loss tangent of 0.0027. Other dimensions of this sensor as given in Table 2 result in two identical resonators working primarily between 2.5–3 GHz. The corresponding active circuit to improve the quality factor of the system is shown in Figure 4b.

**Figure 5:**
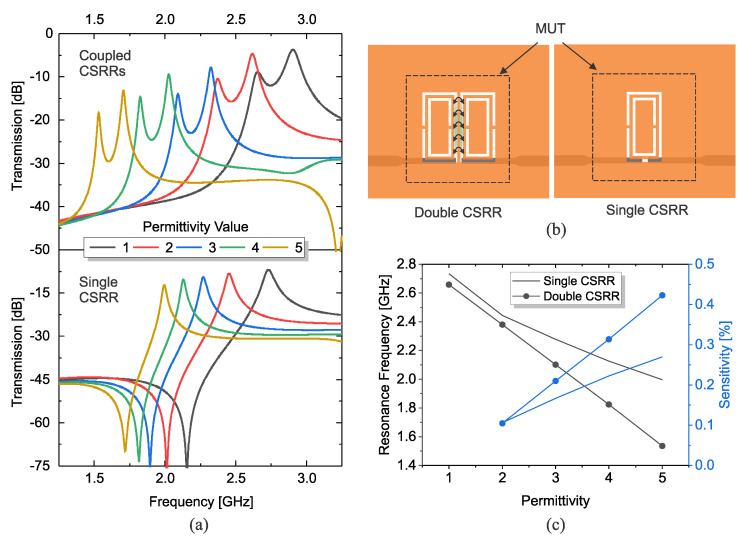
(**a**) Enhanced sensitivity with coupled CSRR in passive mode of sensors (amplifier is not applied). (**b**) Schematic of simulation model. (**c**) Corresponding frequency shift (black) and sensitivity (blue) vs. permittivity.

CSRR resonators are designed with a rectangular geometry to facilitate a high degree of coupling with another analogous resonator via capacitive coupling. A pair of CSRRs are positioned at a distance of 1 mm apart. Hereafter, the configuration of the two resonators aligned in this manner is referred to as coupled complementary split-ring resonators (CCSRRs). This design aims to enhance the sensitivity of the sensor when the coupled region between the two parallel slots is employed to probe the properties of the surrounding material. Investigating the structure of single CSRRs vs. coupled-CSRRs is conducted in the full-wave simulator HFSS as shown in Figure 5a, with a 1.8 mm thick MUT that is demonstrated in Figure 5b with a dashed line. A low permittivity variation is assumed for illustrative purposes, where $\varepsilon_{r}\to \left\{1,2,3,4,5\right\}$. Similar variations for both sensors result in significantly higher shifts in the response of the CCSRR (top) vs. single CSRR (bottom) for both resonance frequencies. The variation in the frequency of operation for the lower resonance frequency $\omega_{1}$ is depicted in Figure 5c, where $\omega_{1}$ CCSRR drops by 1.1 GHz vs. 0.7 GHz for CSRR. This information is also translated to the sensitivity of the proposed sensor types with the following definition:

(15) $$S\left[\%\right]=100\times \frac{\Delta f_{res}}{f_{0}\;\cdot \;\Delta \varepsilon_{r}}$$

where $\Delta f_{res},\;\Delta \varepsilon_{r}$, and $f_{0}$ are change in the resonance frequency, change in the permittivity with respect to air, and initial resonance frequency (bare sensor), respectively [3]. In this analysis, this change of single CSRR to double CSRR translates to a drastic sensitivity enhancement by 70%. This phenomenon can be interpreted with respect to the equivalent circuit model, as shown in Figure 1, as in the coupled CSRR sensor; in addition to the main CSRR capacitor, there is an additional coupling capacitor $C_{m}$ that is affected by the exposed material. This coupling modification results in a net frequency shift according to derivations from (7)–(10). Compared to the single CSRR, wherein only the main capacitor of the CSRR is engaged, the coupling between resonators results in enhanced sensitivity for a given material.

## 5. Loss-Compensation Mechanism

Operation of this amplifier incorporates collector feedback biasing. In this configuration, negative feedback is utilized to inhibit thermal runaway and stabilize the operating point. In this biasing scheme, the base resistor is connected to the collector, rather than being directly connected to the DC source. Consequently, any occurrence of thermal runaway effectively regulates the transistor’s base current.

The main algorithm for designing a regenerative amplifier using CSRR as the tank is thoroughly explained in Algorithm 1.
**Algorithm 1:** Designing a Regenerative Amplifier-Based Active Microwave Resonator.1:Set desired operating central frequency ($(f_{1}+f_{2})/2$)2:Design a passive CCSRR to obtain desired operating frequencies ($f_{1},f_{2}$)3:Select a suitable BJT considering frequency response and noise characteristics $\approx f_{1},f_{2}$4:Design the common emitter amplifier circuit with collector-feedback biasing components to ensure BJT operates in its active region5:Design TLs to feed the CSRR in a two-port configuration:
Calculate the required length and width of the TLs to act as phase shiftersConnect the transmission lines to the CCSRR input and output6:Design a positive feedback network, considering Barkhausen  [3], and connect it from the BJT collector to the resonator input through the phase-shifting TL and close the loop with another phase-shifter TL from CSRR output to the BJT base.7:Optimize feedback network for sufficient loss compensation without self-oscillation8:Verify stability and Q-factor enhancement through simulations on $f_{1}$

**Figure 6:**
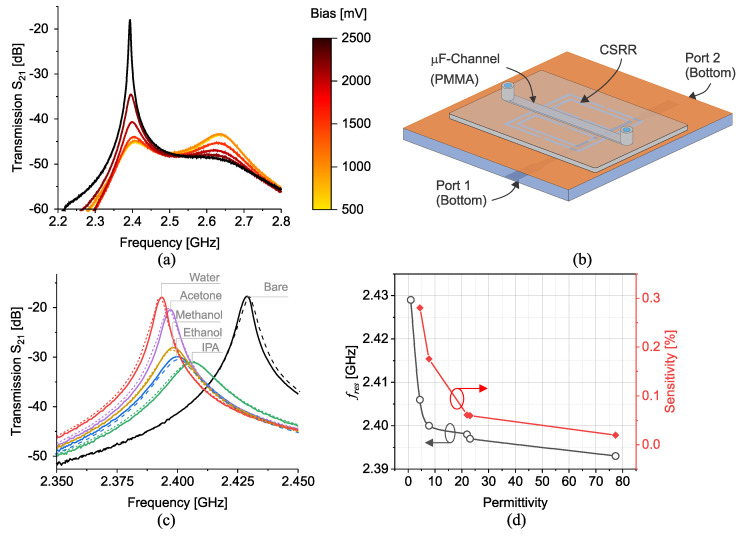
(**a**) Impact of bias voltage on measured transmission ($S_{21}$). (**b**) The schematic of resonator hosting a microfluidic channel. (**c**) Measured (solid line) with $V_{CC}=2.5$ V vs. simulated (dashed line) $S_{21}$ of common chemicals. (**d**) Corresponding measured frequency shifts black and sensitivity red vs. permittivity.

Adhering to the outlined design steps for a target frequency of 2.45 GHz yields the transmission response depicted in Figure 6a. The inclusion of bias voltage $V_{CC}$ serves to regulate the amplifier’s operation. It becomes evident that bias voltages exceeding 500 mV effectively trigger the amplifier. Further increasing of the bias voltage results in loss compensation. For one, the sharpness of the sensor transmission response in air (no MUT) increases from 70 in the passive mode up to and beyond 2700 in active mode with $V_{CC}=2.5$ V. Due to the amplifier’s contribution to the sensor response, this circuit is henceforth referred to as an *active resonator*. The active resonator design is fine-tuned to ensure improved sharpness at the lower frequency as a consequence of optimal phase compensation. Given that the sensor is intended for use as an active resonator, the degree of loss compensation must be carefully controlled to prevent the system from entering an oscillatory regime.

Poly(methyl methacrylate) (PMMA)-based microfluidic channels have emerged as a promising platform for liquid sensing applications due to their excellent microwave transparency, biocompatibility, and ease of fabrication. To enhance the wettability of the channel surface and promote homogeneous fluid flow, a hydrophilization process is employed. Consequently, the modified PMMA microfluidic channels exhibit improved wetting properties, ensuring a stable and consistent liquid flow within the channels. The hydrophilized PMMA-based microfluidic systems are well-suited for a wide range of sensing applications including biological tests, chemical analysis, and environmental monitoring. In this study, a PMMA-based microfluidic channel is utitilzed, which is modeled in simulation as well as found in the Figure 6b, which hosts the injected liquid as the material under test.

The schematic of the proposed CCSRR is simulated in passive form in ANSYSHFSS considering the microfluidic channel hosts various common chemicals including water, acetone, methanol, ethanol, and IPA. The corresponding transmission response of the sensor, which is impacted by the permittivity and loss tangent of the chemicals, is shown in Figure 6c. It is evident that higher loss impedes the peak of $S_{21}$, yet increased permittivity reduces the resonance frequency.

**Figure 7:**
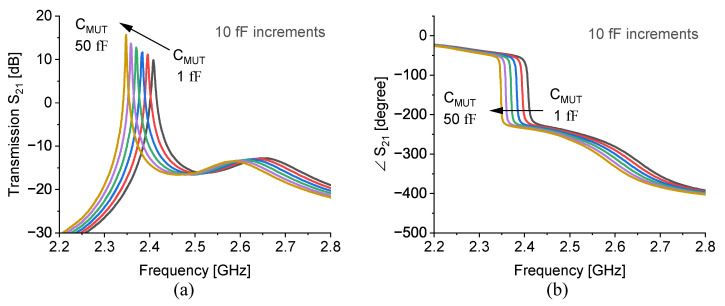
Capacitive loading of CCSRR in ADS simulations (from black (1 fF) to orange (50 fF)) towards obtaining the (**a**) magnitude and (**b**) phase of transmission.

In another study, the contribution of various materials inside the microfluidic channel is simulated in a circuit simulator advanced design system (ADS) to extract the net capacitive impact of each material. In this regard, the sensor is modeled as shown in Figure 6b, yet the microfluidic channel is substituted by a capacitor that is across the adjacent slots of the CCSRR. As a result, a range of capacitance variation is explored that results in roughly the same downshift in the resonance frequency that is achieved by HFSS simulations. Figure 7a demonstrated the magnitude of $S_{21}$ as well as phase variations in Figure 7b demonstrate how the transmission profile undergoes a change when the capacitor value is swept from 1 fF up to 50 fF with 10 fF increments. It is thus clear that the MUT impact on the CCSRR is quite minuscule in magnitude.

**Figure 8:**
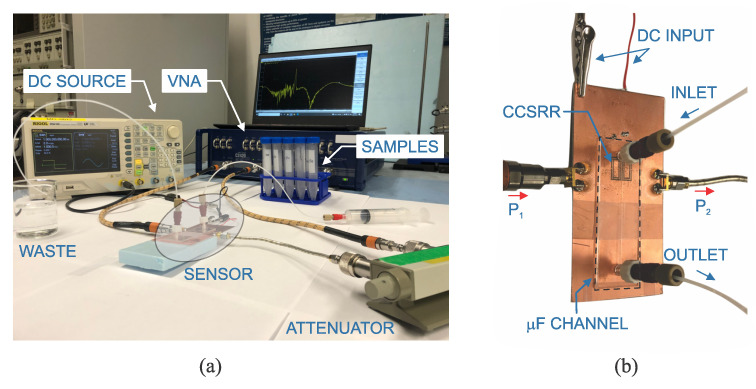
(**a**) Measurement setup with VNA (Vector Network Analyzer), (**b**) Closed-up view of the fabricated active CCSRR.

The sensor is used in the setup shown in Figure 8a, where C2420 VNA measures its transmission, while different samples are injected into the microfluidic channel. The sensor loss is compensated with regenerative feedback with the help of DC source. A closed-up view of the proposed sensor is depicted in Figure 8b and is fabricated on Rogers RO4003 substrate. The fabrication process involves patterning both the top and bottom sides of the substrate using a laser printer and etching unmasked sections with an ammonium persulfate aqueous solution. For this particular design, it is crucial to align the patterns on both sides of the substrate accurately to ensure proper coupling between the resonators and transmission lines of the active circuit. The in-house etching method is followed by soldering surface mount devices onto the substrate.

Next, the performance of the fabricated sensor is evaluated in the context of external liquid interactions. A microfluidic channel, procured from ChipShop, is affixed to the sensor, featuring an embedded channel with a thickness of 150 μm and a width of 2.5 mm, as illustrated in Figure 8b. A 175 μm lid separates the channel from the sensor’s surface. PTFE tubing and fittings facilitate the transport of the injected fluid into and out of the microfluidic channel. The sensor’s input port is connected to an attenuator to mitigate potential high reflections from the loss-compensated sensor back to the network analyzer. All connections utilize phase-stabilized cables to maintain sensitive phase information. Measurement results are regulated through various bias points via $V_{CC}$, which essentially powers the amplifier at different states.

## 6. Material Characterization

In this section, the sensor response is calibrated with respect to the simulation results obtained using common chemicals being injected to the microfluidic channel. The loss compensation of the sensor is accomplished by applying a bias voltage of $V_{CC}=2.1$ V, where the left resonance (refer to Figure 6a) is predominantly compensated. The resonance frequency shifts increase as a consequence of higher permittivity values, illustrating a strong correlation between simulation and measurement relative results. A high congruence is observed when the measured sensor response is compared against the simulated response as shown in Figure 6c with solid lines. The inaccuracy of the sensor response can be assessed from the percentage difference between simulated and measured responses, which is limited to 0.02% on average.

The corresponding frequency shifts are sketched in Figure 6d, which depicts a sharp transition for permittivity values within (1→10), followed by a shallower slope thereafter. The sensitivity of the sensor ($S=\left[f_{0}-f_{res}\right]/\left(f_{0}\;\cdot \;\left(\varepsilon_{r}-1\right))\right)$ [3,15], where $f_{r}es$ represents the resonance frequency due to the material with $\varepsilon_{r}$, and $f_{0}$ denotes the bare resonance frequency, is also illustrated to exhibit ultra-high sensitivities obtained at low effective permittivity changes up to S=0.28 for liquid volume of 4.5 μL.

Furthermore, the dynamic range of the sensor spans a common permittivity range from 1 up to 78, corresponding to a Δf=36 MHz frequency shift. Additionally, the dynamic range of the sensor can be extended by optimizing the coupling, in contrast to a microwave sensor employing only one resonator.

### 6.1. Sensor Response: FAQ (Frequency, Amplitude, Quality-Factor)

Knowing that the proposed sensor is verified with respect to liquids, most of the applications require eliciting the content of a mixture. However, microwave sensors are sensitive to only the effective property of the mixture rather than individual components. This limitation stems from the fact that microwave sensors detect changes in dielectric properties, such as permittivity and conductivity, which are inherently bulk properties of the material system under study. Consequently, when applied to mixtures, microwave sensors provide information that reflects the combined effect of all constituent materials rather than specific contributions from individual components.

**Figure 9:**
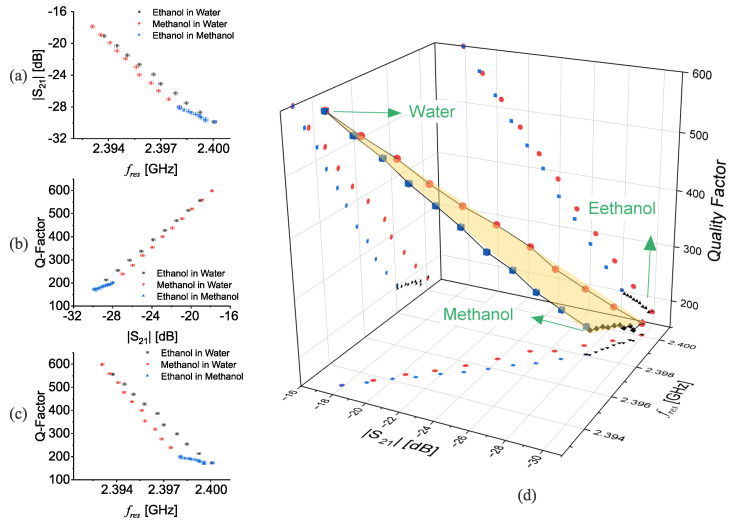
Sensor response for binary mixtures with respect to (**a**) $|S_{21}|$ vs. $f_{res}$, (**b**) Q-factor vs. $|S_{21}|$, (**c**) Q-factor vs. $f_{res}$, (**d**) 3D overview of the sensor calibration surface.

The sensor response in frequency *F*, amplitude *A*, and quality factor *Q* undergoes an effective change as the composition of the mixture varies. However, due to the lack of selectivity, the sensor output (F,A,Q) could be mapped to multiple combinations of several known materials, making it challenging to identify individual constituents within the mixture unambiguously. This phenomenon is known as the “cross-sensitivity” issue, where the sensor’s response may be similar for different material combinations, leading to potential misinterpretation of the measurement data. This shortcoming leads to eliciting more than one parameter at the same time to infer their internal properties. In this regard, water, ethanol, and methanol are chosen as three common liquids to study their binary mixture on the proposed CCSRR. Various concentrations of the mixtures from [0%↦100%] with increments of 10% are measured and $f_{res},{\left|S_{21}\right|}_{@f_{res}}$, and *Q*, are extracted from transmission response, as shown in Figure 9a–c.

It is clear that inspecting only one of these commonly used features in conventional microwave sensing, instead of a one-to-one mapping, one output can be mapped to at least two binary mixtures. This represents how a microwave resonator-based sensor is challenged to recognize correct mixture content. The combinations of all possible binary mixtures are shown in a 3D diagram, as shown in Figure 9d, which suggests a surface that contains all sensing results. Each apex of the triangular surface represents pure chemicals (100%). The surface is also projected to each plane ($|S_{21}|\;vs.\;f_{res}$, $Q\;vs.\;|S_{21}|$, and $Q\;vs.\;f_{res}$). Each point on the boundary of this surface, which can be considered as the *calibration triangle* of the sensor, can be mapped to only one combination of binary mixtures, whereas each point on the yellow surface represents a *ternary mixture*, i.e., mixture of three ingredients.

Following this feature extraction, one can obtain information about the nature of the individual components of a given material in order to efficiently map the extracted sensing parameters (FAQ) to each constituent material.

### 6.2. Generating Training Dataset

In order to design and train a deep neural network (DNN) capable of discerning the nuanced signatures of a proposed complementary split-ring resonator (CCSRR) sensing system, it is imperative to compile a comprehensive dataset that encompasses various permutations of the sensing parameters. In this instance, three sensing parameters are being considered, which correspond to the characteristics of three different liquids: water, methanol, and ethanol. Each of these liquids can be characterized by a unique volumetric quotient, denoted as $V_{1}$, $V_{2}$, and $V_{3}$, respectively.

In order to create a diverse and representative training dataset, 9600 unique combinations of these three liquids are considered. These combinations are achieved by varying the values of the aforementioned volumetric quotients. This variety in the dataset will allow the DNN to recognize a wide range of patterns and relationships, thus improving its predictive accuracy and generalizability.

Once these combinations have been established, the effective permittivity of the binary mixtures of the liquids must be calculated. For this calculation, the Maxwell–Garnett equation is employed, which provides a reliable means of estimating the effective permittivity of a composite material based on the properties of its constituents as follows [51]:

(16) $$\varepsilon_{c}=\varepsilon_{i}\left(1+\frac{3v_{j}\left(\varepsilon_{j}-\varepsilon_{i}\right)}{\left(1-v_{j}\right)\left(\varepsilon_{j}-\varepsilon_{i}\right)+3\varepsilon_{i}}\right)$$

In the context of the Maxwell–Garnett equation, $\varepsilon_{c}$ denotes the effective permittivity of the composite (i.e., the mixture), while $\varepsilon_{i}$ and $\varepsilon_{j}$ represent the permittivities of the individual constituents. The volumetric fractions $v_{i}$ and $v_{j}$ represent the proportions of these constituents in the mixture. It is important to note that $v_{j}=1-v_{i}$, as the total volume of the mixture, must be accounted for by the volumes of its constituents.

An important consideration when applying the Maxwell–Garnett equation in this context is that the permittivities $\varepsilon_{i}$ and $\varepsilon_{j}$ are frequency dependent. This means that they vary according to the frequency of the applied electric field. This frequency dependency adds another layer of complexity to the problem but also presents an opportunity for the DNN to learn more intricate patterns and relationships.

The process of training a deep neural network (DNN) to interpret the sensing parameters of a CCSRR sensor involves the following steps. Firstly, the volumetric ratios of the liquids under examination are converted into equivalent effective permittivities using the Maxwell–Garnett equation. These effective permittivities are considered as the MUT and subsequently simulated using a high-frequency full-wave solver, such as HFSS. This results in the generation of the sensor transmission response or the output data.

**Figure 10:**
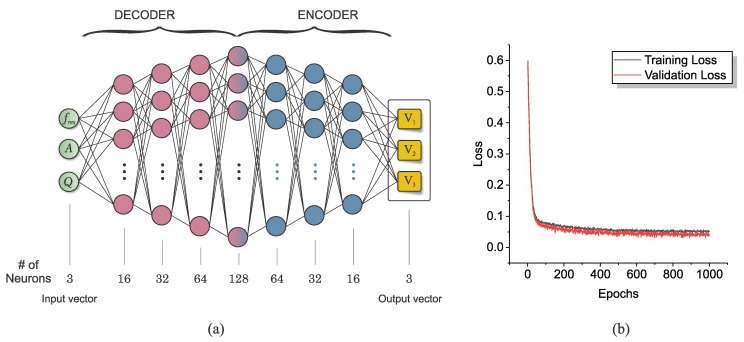
(**a**) Architecture of a deep neural network with a decoder/encoder format. (**b**) Loss function.

To derive the frequency, amplitude, and quality factor (FAQ) parameters, the sensor responses for each case are extracted. This forms the training dataset. It is noteworthy that this dataset is constructed by observing these results in reverse, meaning the output parameters of the sensor (FAQ) serve as the input data, which are to be mapped to the constituent volumetric ratios ($V_{1}$, $V_{2}$, $V_{3}$) as the output, as illustrated in Figure 10a.

### 6.3. Characterizing with a Deep Neural Network

The architecture of the neural network employed in this study comprises an input layer, a series of hidden layers, and an output layer. The input layer takes in a three-feature input data which corresponds to the FAQ parameters. These data are then mapped to a 128-feature data in the middle layer, representing high-feature basis functions. Subsequently, they are mapped into a three-feature output data, reflecting the volumetric ratios. The activation functions of the hidden layers and the output layer are the rectified linear unit (ReLU) functions and the hyperbolic tangent function (tanh), respectively. The performance of the network is assessed based on the mean squared error (MSE) loss function, with the Adam optimizer being employed to minimize this function. To expedite the training process, batch normalization layers are applied before each hidden layer. An early stopping condition, based on the validation loss, is applied instead of setting a maximum number of iterations in the training process.

The dataset generated for training and validation purposes is split into a ratio of 80:20. The validation dataset, randomly selected, is kept entirely separate from the training process. The validation loss is thus a crucial measure of the performance of the trained neural network as it provides an unbiased evaluation of the model’s ability to generalize to unseen data. Figure 10b depicts the training and validation loss functions over the course of the training process, which showcases a significant reduction within the first 40 epochs. Additionally, it is important to note that various network topologies and numbers of hidden layers were examined prior to finalizing the network. Our study indicates that a lower number of hidden layers results in inadequate attenuation of the training and validation loss, whereas a higher number of hidden layers leads to a higher validation loss. This evidence underlines the importance of choosing an appropriate architecture for the DNN, balancing complexity with the capacity to learn and generalize.

The implementation of the microfluidic channel, affixed securely to the sensor, ensures a stable and robust measurement region that aligns well with the simulated model. Accordingly, this simulated input dataset is utilized for training the deep neural network (DNN), which will eventually be tested using an unseen measurement dataset. To assess the performance of the DNN in mixture analysis, solutions comprising varying concentrations of ethanol, methanol, and water are mixed and introduced to the fabricated sensor, as depicted in Figure 8a. The frequency, amplitude, and quality factor (FAQ) parameters obtained from the measured sensor responses are then input to the pre-trained DNN, yielding outputs in the form of the volumetric ratios $V_{1}$, $V_{2}$, and $V_{3}$. It is assumed that the concentrations of methanol and water are rigorously controlled at given values, while the remainder of the volumetric ratio is occupied by the third liquid, ethanol. This is illustrated in Table 3. For instance, if water comprises 10% and methanol takes 30% of the mixture, the remaining 60% must be occupied by ethanol. In this scenario, the DNN evaluates the ethanol concentration to be 58.1%, which has 1.9% inaccuracy.

It is worth noting that for each mixture, the measurement error inherent in the input FAQ parameters results in a range of output volumetric ratios. The DNN also provides an estimate of the absolute maximum error, |e|, which denotes how much the mapped predicted concentration fluctuates due to incertitude in the input FAQ measured values. It is observed that the measurement results are generally more accurate with closer average concentrations to the desired value for binary mixtures, i.e., mixtures involving only two components. However, the accuracy tends to decrease when a third solvent is introduced, where maximum inaccuracy is assigned with the sample consisting of methanol:water:ethanol with 20%:30%:50%, which suggests 4.3% average incertitude.

This prompts the consideration of alternative characterization mechanisms, such as convolutional neural networks (CNN). CNNs, which have been extensively used in image processing and analysis, can potentially offer a more sophisticated approach to mixture analysis, as they are capable of identifying complex patterns in multidimensional data.

### 6.4. Convolutional Neural Network

**Figure 11:**
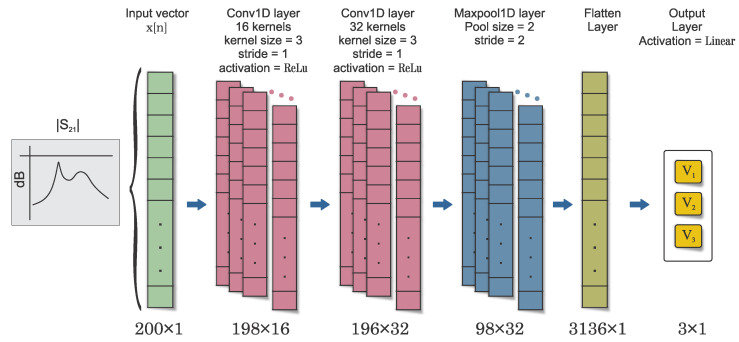
Architecture of convolutional-neural-network-based regression for three concentration of $V_{1},\;V_{2},\;V_{3}$.

CNNs, owing to their hierarchical structure and ability to extract robust high-level features, have demonstrated strong learning capabilities and generalization performance, making them well-suited to this task. The proposed CNN architecture comprises two 1D convolution layers, one 1D max pooling layer, one flatten layer with dropout, and a final dense or fully connected layer with softmax or sigmoid activation for classification output, as shown in Figure 11. The raw one-dimensional sensor transmission response, denoted as x[n], is fed as input to the first convolutional layer. The convolutional layers use filters or kernels to produce feature maps by convolving with the input signal. The number and size of kernels are key for capturing relevant features from the signal. The output of each convolution operation is calculated using a specific equation, where the activation function is applied to the sum of bias and the convolution of the previous layer’s features and the current layer’s kernels. The first convolution layer contains 16 convolution kernels, and the second convolution layer contains 32 kernels. Each kernel has a size of v = 3 and a stride of 1. The filter weights are initialized using the He uniform initializer [61], and the bias vector is initialized to all zeros. This operation is performed for each filter in both layers, resulting in 16 outputs for the first convolution layer and 32 outputs for the second convolution layer. The activation function used is the rectified linear unit (ReLU), which introduces nonlinearity into the model and is defined as the maximum of 0 and the input. This function is known to accelerate the convergence of stochastic gradient descent compared to other functions.

The flattened output is processed by a subsequent dense or fully connected layer, which is responsible for perform a regression on all three variables $V_{1},\;V_{2},\;V_{3}$. The operation of this layer can be mathematically represented as follows:

(17) $$\mathrm{output}=\sigma (<\mathrm{input},\Omega_{d}>+b_{d})$$

In this equation, $<\mathrm{input},\Omega_{d}>$ denotes the dot product of the input vector and the weight matrix $\Omega_{d}$ associated with this layer, $b_{d}$ represents the bias vector for this layer, and σ stands for the activation function, which is a linear one (i.e., identity function). No further normalization is needed as the output is not a probability distribution but a single value. It should be noted that the cost function used for training the network is mean squared error (MSE), calculated over all output variables as explained in Appendix A.

The effectiveness of the CNN approach is also evaluated using the same data responses, with results compared to those from the previous ANN model. The CNN was trained using the same training set as the ANN. A notable difference between the ANN and the CNN implemented in this study pertains to the size of the input vector. The ANN was restricted to three feature quantities (FAQs), whereas the CNN accommodated a significantly larger vector comprising transmission responses across 1001 frequency points.

Table 4 presents measurement responses similar to those in Table 3, although the results for mixtures of three constituents show considerable improvement in the CNN model such that maximum average concentration deviates by only 0.7% for the sample of methanol:water:ethanol of 20%:30%:50%. This enhanced performance in measurement responses suggests that supplying the network with a larger volume of input data enhances the accuracy of the predictions. In our case, the input data correspond to sensor responses at varying frequencies, which provides a more comprehensive characterization of the material properties across a broader frequency range rather than at a single frequency point.

By facilitating the processing of larger input vectors, the CNN can harness additional sensor response data at multiple frequencies to improve the accuracy of material characterization. Therefore, the use of CNNs offers significant advantages for tasks requiring the analysis of complex mixtures with multiple constituents.

## 7. Conclusions

In this study, we propose an artificial intelligence (AI)-assisted algorithm as a promising strategy to enhance the ability of microwave sensors to detect and characterize mixtures. We design a highly sensitive microwave sensor operating at 2.45 GHz, centered on coupled complementary split ring resonators (CSRRs). The sensor’s resolution is substantially improved by mitigating loss content using a regenerative amplifier. The region between the CSRRs, where coupling occurs, is leveraged as the sensing region. In a binary liquid mixture characterization, an active sensor is employed that boasts nearly a ten-fold enhancement in the sensor’s quality factor. The frequency, amplitude, and quality factor of the transmission response are extracted and mapped to the corresponding volumetric ratios of the constituent materials using a deep neural network. However, this method primarily demonstrated success for binary mixtures. To ascertain the mixture ratios of ternary solutions, we employed a more advanced machine learning algorithm, the convolutional neural network (CNN). A significant improvement in the analysis of sensor response was achieved when the entire transmission response was inputted to the CNN. This approach offers a promising solution to the selectivity limitations commonly encountered with microwave sensors, thereby indicating the potential of this AI-assisted method to advance microwave sensor technology for mixture characterization.

## Figures and Tables

**Table 1:** Debye parameters of water, methanol, and ethanol at 25 °C.

Parameters	$\varepsilon_{\infty }$	$\varepsilon_{0}$	$\tau_{0}$ [ps]
**Water**	4.9	78.4	8.23
**Methanol**	5.3	32.6	46.8
**Ethanol**	3.9	23.7	145.1

**Table 2:** Table of design parameters. All dimensions are in [mm].

**Parameter**	***W***	***g***	$L_{1}$	$L_{2}$	***s***
**Value**	0.5	0.5	3	9	0.5
**Parameter**	$W_{\mathrm{TL}}$	$S_{\mathrm{TL}}$	$L_{\mathrm{TL}}$	$g_{\mathrm{TL}}$	$W_{\mathrm{TL}}$
**Value**	1	1	11.5	1	1

**Table 3:** Predicted concentration of ethanol with known values for methanol/water using ANN.

Water [%]	Methanol [%]											
	0		10		20		30		40		50	
	Avg.	max|e|	Avg.	max|e|	Avg.	max|e|	Avg.	max|e|	Avg.	max|e|	Avg.	max|e|
0	99.1	0.4	88.5	0.7	80.5	0.4	68.9	0.5	59.1	0.3	49.2	0.2
10	91.2	0.4	77.2	0.7	74.1	0.5	58.1	0.4	52.7	0.2		
20	79.4	0.5	64.4	0.4	56.9	0.3	53.7	0.2				
30	70.4	0.5	64.3	0.2	45.7	0.6						
40	58.9	0.5	53.1	0.4								
50	51.1	0.6										

**Table 4:** Predicted concentration of ethanol with known values for methanol/water using CNN.

Water [%]	Methanol [%]											
	0		10		20		30		40		50	
	Avg.	max|e|	Avg.	max|e|	Avg.	max|e|	Avg.	max|e|	Avg.	max|e|	Avg.	max|e|
0	99.5	0.5	89.7	0.6	79.5	0.4	69.7	0.6	60.1	0.7	50.4	0.6
10	90.5	0.4	79.5	0.7	70.2	0.6	59.6	0.4	49.7	0.3		
20	80.4	0.5	69.7	0.3	59.7	0.5	50.4	0.5				
30	69.7	0.6	60.2	0.3	49.3	0.4						
40	59.5	0.4	50.4	0.7								
50	49.8	0.4										

## Data Availability

Not applicable.

## Appendix A

In the context of predicting three continuous parameters using a regression-based convolutional neural network (CNN), the mean squared error (MSE) loss function serves as an effective measure of the average squared difference between the true and predicted values of the three parameters.

Suppose we have *n* samples, and for each sample *i*, we have the predicted output values $y_{{\mathrm{pred}}_{i}}=\left[y_{1,{\mathrm{pred}}_{i}},y_{2,{\mathrm{pred}}_{i}},y_{3,{\mathrm{pred}}_{i}}\right]$ and the true output values $y_{{\mathrm{true}}_{i}}=\left[y_{1,{\mathrm{true}}_{i}},y_{2,{\mathrm{true}}_{i}},y_{3,{\mathrm{true}}_{i}}\right]$. The MSE for this multi-output regression problem can be expressed as

(A1) $$MSE=\frac{1}{n}\sum_{i=1}^{n}\left[{\left(y_{1,{\mathrm{pred}}_{i}}-y_{1,{\mathrm{true}}_{i}}\right)}^{2}+{\left(y_{2,{\mathrm{pred}}_{i}}-y_{2,{\mathrm{true}}_{i}}\right)}^{2}+{\left(y_{3,{\mathrm{pred}}_{i}}-y_{3,{\mathrm{true}}_{i}}\right)}^{2}\right]$$

This formula calculates the sum of the squared differences between the predicted and actual values for each of the three parameters across all samples in the dataset. The sum is then divided by the total number of samples *n* to derive the average.
